# Open transcervical diverticulectomy for recurrent Zenker’s diverticulum after Z-POEM: a case report

**DOI:** 10.1093/jscr/rjag532

**Published:** 2026-06-26

**Authors:** Ahmed Rayyan, Murad Isaak Alshamisti, Nouraldeen Deeb, Issa Abu Iram, Bashar Douden, Ahmad Almasalmah, Samer Amayre, Anas Alasafrah

**Affiliations:** Faculty of Medicine, Al-Quds University, Abu Dis Campus, PO Box 20002, Postal Code 20002, Abu Dis, Jerusalem Governorate, Palestine; Faculty of Medicine, Al-Quds University, Abu Dis Campus, PO Box 20002, Postal Code 20002, Abu Dis, Jerusalem Governorate, Palestine; Faculty of Medicine, Al-Quds University, Abu Dis Campus, PO Box 20002, Postal Code 20002, Abu Dis, Jerusalem Governorate, Palestine; Faculty of Medicine, Al-Quds University, Abu Dis Campus, PO Box 20002, Postal Code 20002, Abu Dis, Jerusalem Governorate, Palestine; Faculty of Medicine, Al-Quds University, Abu Dis Campus, PO Box 20002, Postal Code 20002, Abu Dis, Jerusalem Governorate, Palestine; Faculty of Medicine, Al-Quds University, Abu Dis Campus, PO Box 20002, Postal Code 20002, Abu Dis, Jerusalem Governorate, Palestine; Faculty of Medicine, Al-Quds University, Abu Dis Campus, PO Box 20002, Postal Code 20002, Abu Dis, Jerusalem Governorate, Palestine; Thoracic Surgery Department, Al-Ahli Hospital, PO Box 224, Postal Code 22500, Ras Al-Joura District, Hebron, Palestine; General Surgery Department, Faculty of Medicine, Al-Quds University, Abu Dis Campus, PO Box 20002, Postal Code 20002, Abu Dis, Jerusalem Governorate, Palestine

**Keywords:** Zenker’s diverticulum, Z-POEM, recurrent diverticulum, open transcervical diverticulectomy, cricopharyngeal myotomy, case report

## Abstract

Zenker’s diverticulum is increasingly treated with endoscopic techniques, including peroral endoscopic myotomy (Z-POEM), but persistent or recurrent symptoms may still occur. We report a 77-year-old woman with longstanding dysphagia, regurgitation of undigested food, cough, and aspiration events despite prior endoscopic septotomy/Z-POEM. Repeat evaluation with barium swallow, computed tomography, and endoscopy confirmed a persistent large Zenker’s diverticulum with retained contents. Given severe symptoms, aspiration risk, prior endoscopic failure, and altered cervical anatomy after previous thyroid surgery, she underwent open left transcervical diverticulectomy with cricopharyngeal myotomy. Recovery was uneventful apart from transient hoarseness, and follow-up showed marked improvement in swallowing and resolution of regurgitation and aspiration. This case highlights the importance of repeat imaging in symptomatic patients after Z-POEM and supports open transcervical repair as an effective salvage option in selected cases.

## Introduction

Zenker’s diverticulum is a pulsion diverticulum arising through Killian’s dehiscence because of impaired upper esophageal sphincter opening and cricopharyngeal dysfunction. It usually affects older adults and may cause dysphagia, regurgitation of undigested food, chronic cough, halitosis, and aspiration. Barium swallow remains central to diagnosis because it defines pouch size and demonstrates functional retention [1, 2].

Although flexible endoscopic septotomy and Z-POEM are less invasive alternatives to open surgery, persistent or recurrent symptoms remain clinically important, particularly with large diverticula, incomplete septal division, or complex cervical anatomy. Open diverticulectomy with cricopharyngeal myotomy therefore remains relevant as a definitive salvage option after failed endoscopic therapy [1, 3–6].

## Case presentation

A 77-year-old woman with hypertension and previous total thyroidectomy followed by reoperation for recurrent thyroid enlargement in 2014 and 2016 presented with an 8-year history of progressive dysphagia, regurgitation of undigested food, nocturnal cough, heartburn, halitosis, and vomiting of retained food. Symptoms were worse with solids and when lying down. She had reduced oral intake because of fear of coughing and regurgitation and had recurrent chest infections consistent with aspiration pneumonia requiring hospitalization for intravenous antibiotics.

Symptoms began in 2017, when upper endoscopy showed a Zenker’s diverticulum of about 3 cm. She underwent endoscopic septotomy/Z-POEM with only slight early improvement, followed by gradual recurrence and progression. This clinical course suggested persistent or incompletely treated disease rather than definite *de novo* recurrence, although the mechanism could not be proven because the original procedural details were unavailable. Possible contributors included incomplete septal or cricopharyngeal myotomy, residual pouch retention, progressive pouch distension, and anatomical complexity after previous cervical operations.

**Figure 1:**
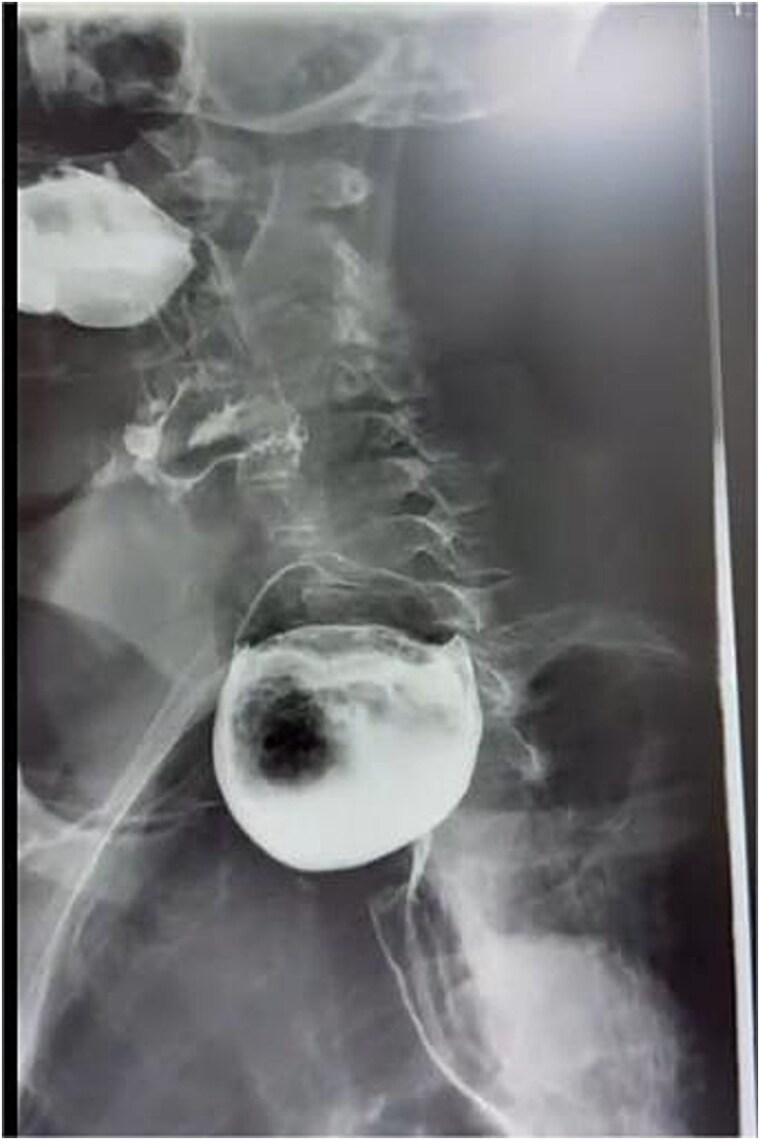
Barium swallow (lateral view) demonstrating a large contrast-filled posterior pharyngoesophageal diverticular pouch with an air-contrast level, consistent with Zenker’s diverticulum.

**Figure 2:**
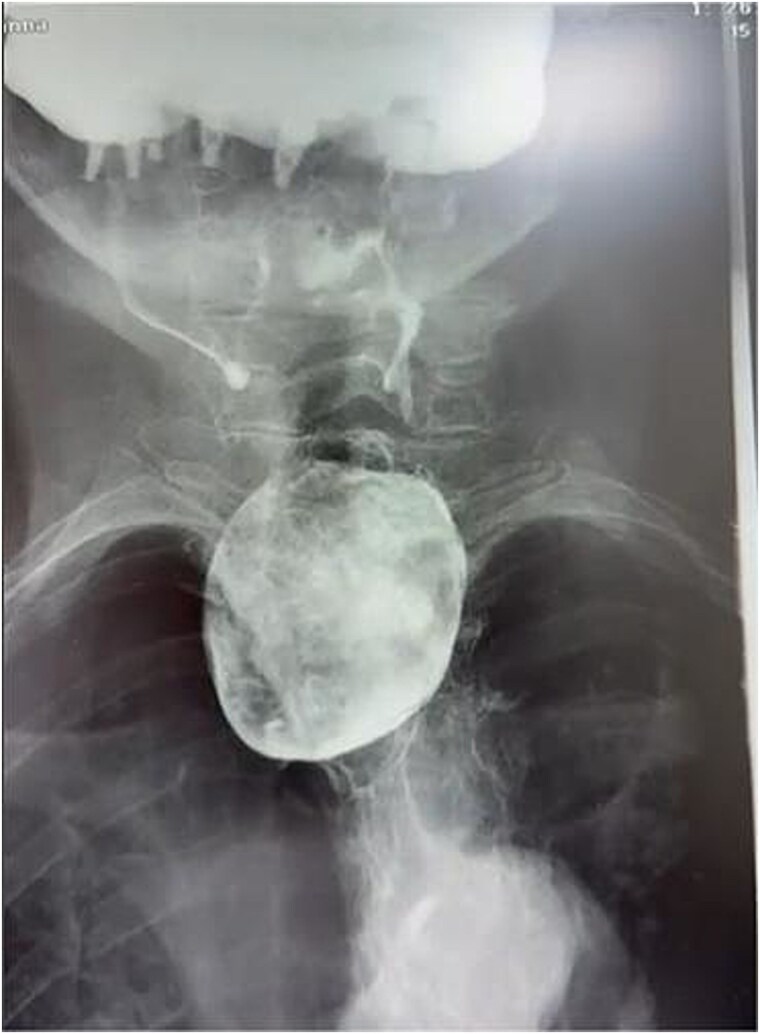
Barium swallow (anteroposterior view) demonstrating a large contrast-filled proximal diverticular pouch at the thoracic inlet.

**Figure 3:**
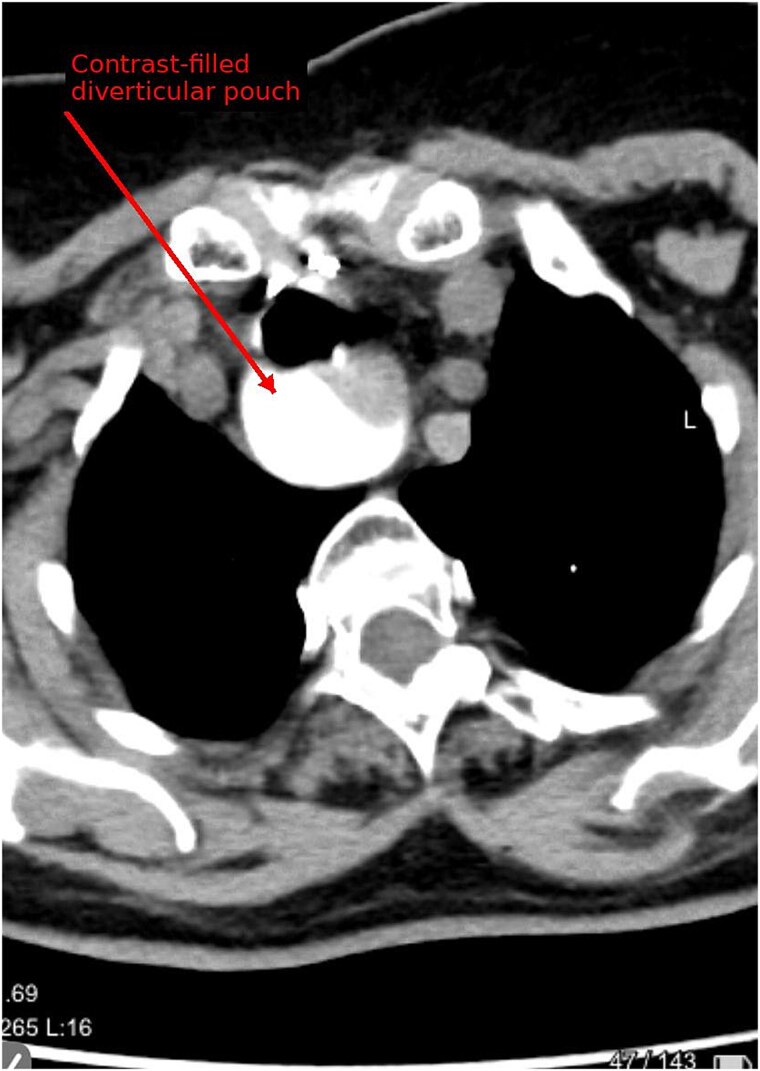
Contrast-enhanced CT (axial) demonstrating a contrast-opacified posterior pharyngoesophageal outpouching consistent with Zenker’s diverticulum.

**Figure 4:**
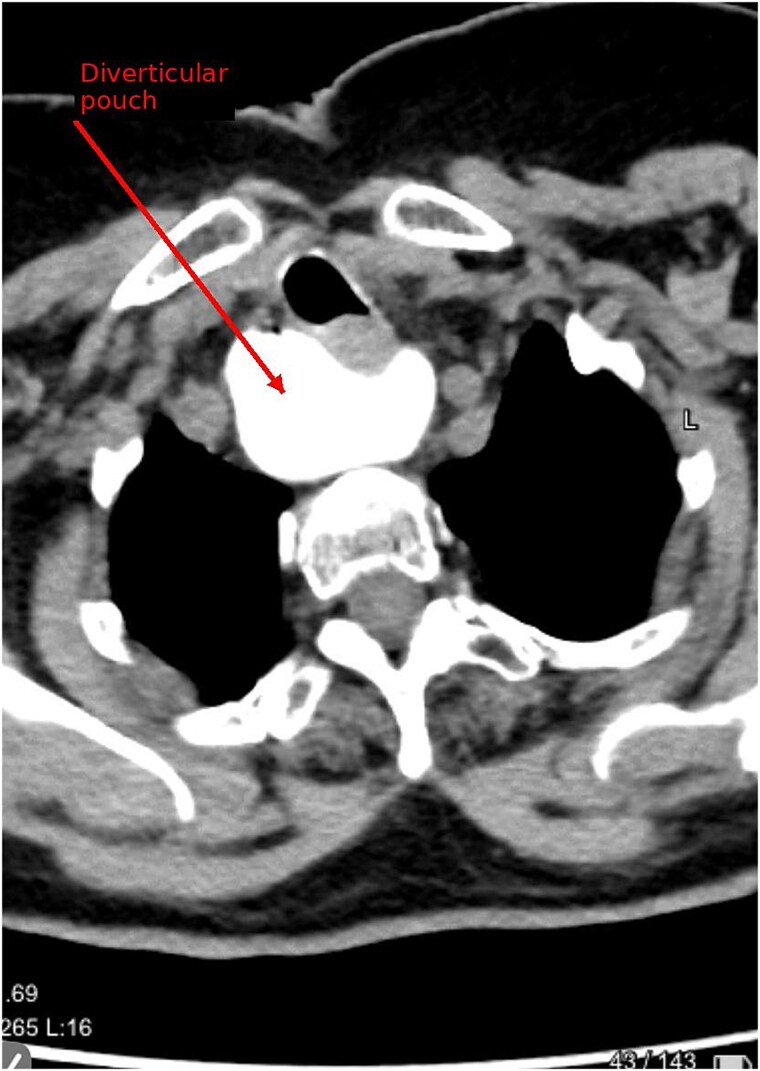
Contrast-enhanced CT (axial) demonstrating the diverticular pouch at the thoracic inlet level.

**Figure 5:**
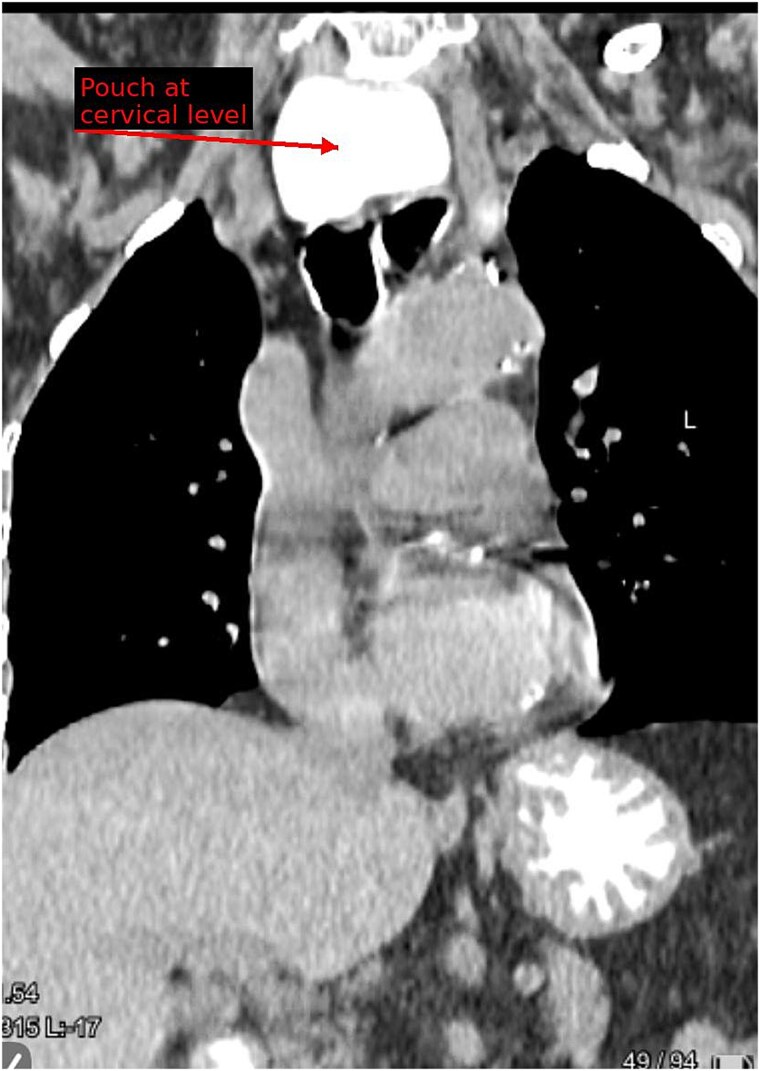
Contrast-enhanced CT (coronal reconstruction) demonstrating the cervical-level pouch and its craniocaudal extension.

Repeat barium swallow in October 2025 showed a large posterior proximal pharyngoesophageal pouch measuring ~6.5 × 5 × 4 cm. (Figs 1 and 2) The pouch filled immediately and remained markedly contrast-filled after 30 min, with complete emptying only on next-day delayed imaging, indicating pronounced retention. Repeat endoscopy in November 2025 again showed a diverticulum, reported as about 3 cm. Contrast-enhanced computed tomography (CT) demonstrated a posterior contrast-opacified pharyngoesophageal outpouching at the thoracic inlet, measuring ~1.7 × 3.6 × 4.0 cm and extending from C7 to T4, without perforation or surrounding inflammation (Figs 3–5). The size differences were interpreted as reflecting variable distension and filling between studies.

After acceptable preoperative cardiopulmonary assessment, she underwent elective open left transcervical diverticulectomy with an ~3-cm cricopharyngeal myotomy in November 2025. Intraoperative endoscopy confirmed a large diverticulum containing retained food, which was evacuated. The pouch was resected with a stapling device and reinforced with sutures; leak testing was negative, and a Jackson–Pratt drain was placed. Histopathology confirmed Zenker’s diverticulum without dysplasia or malignancy. Postoperatively, she developed transient hoarseness that resolved over ~2 months. At 2.5-month follow-up, regurgitation and aspiration had resolved and she tolerated a normal diet.

## Discussion

This case shows that persistent symptoms after Z-POEM require objective reassessment rather than attribution to nonspecific dysphagia. In this patient, the esophagram was especially informative because it demonstrated immediate filling, marked retention, and delayed emptying of a large pouch, functional findings not fully captured by endoscopy alone [1, 5, 6].

The most plausible explanation was persistent or incompletely treated disease, supported by the limited initial response, progressive symptoms, and retention-dominant pouch. However, true recurrence cannot be excluded without the original operative details. The discrepancy between endoscopic, fluoroscopic, and CT measurements should not be viewed as contradictory because Zenker’s diverticula are dynamic structures whose apparent size depends on distension, retained contents, and the phase of swallowing or filling. Differentiation from other cervical esophageal diverticula, especially Killian–Jamieson diverticulum, may also be difficult; in this case, the posterior origin and operative anatomy supported Zenker’s diverticulum [2, 7].

Repeat endoscopic therapy may be reasonable for a clearly residual septum, smaller or moderate pouch, favorable exposure, limited aspiration risk, or high operative risk. Open surgery may be preferred when there is a large retention-dominant pouch, severe aspiration, uncertain anatomy, suspected fibrosis after prior therapy, or need for definitive pouch excision. Open transcervical diverticulectomy with cricopharyngeal myotomy provides direct exposure, removal of the reservoir, and formal myotomy, although it carries risks including recurrent laryngeal nerve injury, leak, wound complications, and longer recovery. In our patient, prior endoscopic failure, recurrent aspiration, delayed pouch emptying, thoracic inlet extension, and previous cervical surgery favored an open salvage approach [3–6].

The favorable outcome supports open repair as an effective salvage option in selected patients. The limitations of this report include its single-case design, short follow-up, and inability to determine the exact mechanism of initial Z-POEM failure.

## Conclusion

Recurrent or persistent Zenker’s diverticulum should be considered when dysphagia, regurgitation, cough, or aspiration continue after Z-POEM. Repeat contrast imaging can reveal functional severity and retention despite variable endoscopic findings. In selected patients with a large retention-dominant pouch, aspiration risk, failed endoscopic therapy, or complex cervical anatomy, open transcervical diverticulectomy with cricopharyngeal myotomy remains an effective salvage treatment.

## Data Availability

The raw data supporting the conclusions of this article will be made available by the authors, without undue reservation.
